# Preserved Coupling between the Reader's Voice and the Listener's Cortical Activity in Autism Spectrum Disorders

**DOI:** 10.1371/journal.pone.0092329

**Published:** 2014-03-24

**Authors:** Catherine Clumeck, Sarah Suarez Garcia, Mathieu Bourguignon, Vincent Wens, Marc Op de Beeck, Brice Marty, Nicolas Deconinck, Marie-Vincianne Soncarrieu, Serge Goldman, Veikko Jousmäki, Patrick Van Bogaert, Xavier De Tiège

**Affiliations:** 1 Laboratoire de Cartographie fonctionnelle du Cerveau, UNI – ULB Neuroscience Institute, Université libre de Bruxelles (ULB), Brussels, Belgium; 2 Laboratoire de Recherches Psychiatriques, UNI – ULB Neuroscience Institute, Université libre de Bruxelles (ULB), Brussels, Belgium; 3 Centre de référence des troubles envahissants du développement et des troubles autistiques, Hôpital Universitaire des Enfants Reine Fabiola (HUDERF), Brussels, Belgium; 4 Brain Research Unit, O.V. Lounasmaa Laboratory and MEG Core, Aalto NeuroImaging, Aalto University, Espoo, Finland; University College of London - Institute of Neurology, United Kingdom

## Abstract

**Purpose:**

Investigating the steadiness of the phase-coupling between the time-course of the reader's voice and brain signals of subjects with autism spectrum disorder (ASD) passively listening to connected speech using magnetoencephalography (MEG). In typically developed subjects, such coupling occurs at the right posterior temporal sulcus (pSTS) for frequencies below 1 Hz, and reflects the neural processing of sentence-level rhythmic prosody at the prelexical level.

**Methods:**

Cortical neuromagnetic signals were recorded with MEG (Elekta Oy, Finland) while seven right-handed and native French-speaking ASD subjects (six males, one female, range: 13–20 years) listened to live (*Live*) or recorded (*Recorded*) voices continuously reading a text in French for five minutes. Coherence was computed between the reader's voice time-course and ASD subjects' MEG signals. Coherent neural sources were subsequently reconstructed using a beamformer.

**Key findings:**

Significant coupling was found at 0.5 Hz in all ASD subjects in *Live* and in six subjects in *Recorded*. Coherent sources were located close to the right pSTS in both conditions. No significant difference was found in coherence levels between *Live* and *Recorded*, and between ASD subjects and ten typically developed subjects (right-handed, native French-speaking adults, 5 males, 5 females, age range: 21–38 years) included in a previous study.

**Significance:**

This study discloses a preserved coupling between the reader's voice and ASD subjects' cortical activity at the right pSTS. These findings support the existence of preserved neural processing of sentence-level rhythmic prosody in ASD. The preservation of early cortical processing of prosodic elements in verbal language might be exploited in therapeutic interventions in ASD.

## Introduction

Autism spectrum disorders (ASD) represent an heterogeneous group of neurodevelopmental disorders of variable severity characterized by clinical features that can be classified into two main groups: social/communication disorders and narrow interest/stereotyped-repetitive behaviors (for a review, see [1]). While other neuropsychiatric disorders can share some of these symptoms, the precocity and the pervasive characteristic of social and communication impairments are highly specific of ASD (for a review, see [2]).

Based on structural and functional neuroimaging studies, several brain areas seem to play a major pathophysiological role in ASD social and communication impairments (for a review, see [3]). Among them, the cortical structures around the posterior superior temporal sulcus (pSTS) are of particular interest as they play a key functional role in social cognition, and are structurally and functionally abnormal in ASD subjects (for a review, see [4]). Indeed, several studies pointed out the involvement of the pSTS in the visual analysis of social cues such as gaze orientation or body movements (for a review, see [5]). Accumulating evidence also reveals that the pSTS is implicated in social perception through the auditory analysis of vocal and non-vocal sounds (for reviews, see [4], [6]). Functional neuroimaging studies have actually demonstrated the existence of voice-selective regions in both pSTS [7]. Based on these data, some authors suggest that a major functional role of the pSTS is to parse auditory or visual inputs into discrete units in order to extract their meaning [6]. Finally, this brain area was also identified as being part of cortical networks supporting theory of mind (for a review, see [8]) and mirror neuron (for reviews, see [9]–[11]) processes; such processes being impaired in ASD subjects [12]–[15]. In addition, several structural and functional neuroimaging studies have shown pSTS abnormalities in ASD such as decreased gray matter concentration in the cortex around the pSTS [16]–[18], rest hypoperfusion [4], [19], and abnormal activation during social tasks or vocal sound processing [20]–[22]. Taken together, these data suggest that the pSTS plays a key role in the pathophysiology of social and communication impairments found in ASD. Several authors thus hypothesize that anomalies involving the pSTS region during early brain development could represent the first step in the cascade of neural dysfunctions underlying ASD [4].

In humans, successful speech comprehension involves segmentation of speech signals into various timescales or temporal windows (TW), so that the spectrotemporal information is concurrently extracted within separate neural streams for speech perception and then integrated in brain regions involved in subsequent lexical computations for speech comprehension (for a review, see [23]). Up to now, two timescales have been investigated in depth in the context of speech perception: the 20–50 ms TW corresponding to the duration of phonemes, and the 150–300 ms TW corresponding to the duration of syllables and some prosodic phenomena (for a review, see [24]). Longer TWs, at the level of the second(s), are involved in sentence-level information [24]. In the context of an ecological continuous listening condition, we recently identified a novel coupling phenomenon at the sentence level, referred to as cortico-vocal coherence (CVC), between the time-course of the reader's voice and the listener's brain signals [25]. Significant coherence or phase coupling below 1 Hz was observed in all subjects within right pSTS and posterior superior temporal gyrus (pSTG) for the speech conditions, and within supra-temporal auditory cortices only for the non-speech vocal condition. In addition, significant coupling at 4–6 Hz (syllable level) was observed in 40% of the subjects at the supratemporal auditory cortex bilaterally. The coupling observed below 1 Hz corresponded to the neural integration of rhythmic prosody at the sentence level within right temporal voice sensitive areas; rhythmic prosody corresponding here to natural pauses occurring in the text in relation to punctuation marks or pauses associated with reader's breathing. These data suggested that right, non dominant, temporal voice sensitive areas contain neurons preferentially integrating sentence rhythmic prosody (TW>1 second) during natural speech perception reflected in rhythmic neuronal activity fluctuations below 1 Hz. Considering the key role of the right pSTS in inter-individual communicative behaviors and the importance of pauses or silences to guide successful verbal conversation [26], the strong reader–listener coupling at the right pSTS observed in this listening setting, was also interpreted as potentially corresponding to the neural processes involved in the recognition of relevant transitions in the speaker's discourse. These neural processes might serve as cues to drive the listener to initiate speech during interactional verbal communication or turn-taking transitions. Interestingly, turn-taking ability is known to be impaired in ASD with reasonable speech development (for a review, see [27]). This impairment is considered to be associated with pragmatic language deficits such as difficulties in sustaining conversation and turn-taking transitions, or in prosody perception [27]. Prosody, which is defined by the suprasegmental features of speech (variation in pitch, intonation, stress, rate, rhythm, duration, pausing, and loudness), facilitates speech comprehension and interindividual verbal communication (for a review, see [28]). While deficits in prosody appear not to be universal in ASD, atypical use of prosody is considered as a hallmark of speakers with such disorders [29]. Furthermore, several behavioral, neurophysiological and neuroimaging data have reported impairments in the production and the perception of prosody in ASD (for reviews, see [28], [30]).

Therefore, considering the putative dysfunction of the pSTS in ASD and the impairments in prosodic perception and pragmatic language in subjects with this disorder, this study aims at investigating the steadiness of the coupling between the time-course of the reader's voice and ASD subjects' brain signals using magnetoencephalography (MEG) and the CVC approach. We predict that pSTS dysfunction in ASD might be reflected in a weaker coupling in ASD subjects, or that the location of coherent brain areas will differ from that observed in typically developed subjects. Conversely, if coupling is unaffected in ASD, it might represent a theoretical support for therapeutic interventions based on low-frequency prosodic content of language to foster social communication in this disorder.

## Methods

### Participants

ASD subjects' clinical details are summarized in Table 1.

**Table 1:** Patients' clinical details.

								Mean
Subjects	1	2	3	4	5	6	7	
Age	18	20	19	15	14	13	15	16
Sex	M	M	M	M	M	F	M	
Diagnosis	AD	AD	AD	ASD	AD	ASD	AD	
Weschler Scales (mean = 10, SD = 3)								
Block design	13	12	3	4	9	12	7	8,5
Similarities	8	9	11	14	8	19	5	10,5
Matrix reasoning	13	12	8	5	10	8	8	9,1
Comprehension	6	5	6	9	1	14	3	6,2
TLOCC								
Comprehension (mean = 57,8; SD = 12,1)	48	58	38	62	22	68	44	48,5
Expression (mean = 46,1; SD = 14,4)	20	42	27	58	14	68	24	36,1
ADOS								
Communication (cut-off for autism = 3)	4	6	5	5	5	5	6	5,1
Social interaction (cut-off for autism = 6)	11	14	9	11	9	7	12	10,4
Total C+SI (cut-off for autism = 10)	15	20	14	16	14	12	18	15,5
Speech abnormalities* (0 = normal, 2 = speech clearly abnormal)	2	2	2	1	2	1	2	1,7

(M: male; F: female; AD: autistic disorder; ASD: Asperger syndrome; SD: standard deviation; TLOCC: test de langage oral complexe pour collégiens; ADOS: autism diagnostic observation schedule; C: communication; SI: social interaction;

*: speech abnormalities associated with autism, coded item 2 of the ADOS Language and Communication domain).

Seven right-handed and native French-speaking subjects (6 males and 1 female, age range: 13–20 years, mean age: 16, normal audition) diagnosed with Asperger Syndrome (2 patients) or autistic disorder (5 patients) were included in the study. The handedness was assessed with the Edinburgh handedness Inventory [31]. The intellectual functioning was assessed by an abbreviated version of the Wechsler Intelligence scale for Children (Fourth Edition) [32] or the Wechsler Adult Intelligence Scale (Third Edition) [33]. The ASD was diagnosed according to DSM-IV criteria [34] and subsequently confirmed by the Autism Diagnostic Observation Schedule-Generic (ADOS) [35]. Based on ADOS, all subjects had weak (2 subjects) or clear (5 subjects) expressive prosodic disorder. To complete the assessment of the expressive language already explored with the ADOS, a brief assessment of the ASD subjects' lexical level was conducted with the *Test de Langage Oral Complexe pour Collégiens* (TLOCC) [36]. Subjects with neurological comorbidities or taking psychotropic treatment were excluded from the study except for one subject who was taking quetiapine (200 mg) for sleep disorder.

The study had the prior approval by the ULB-Hôpital Erasme Ethics Committee. The ASD subjects and their parents gave written informed consent before participating to the study.

Data obtained in the ten right-handed and native French-speaking typically developed healthy adult subjects (range 21–38 years; mean age: 25 years; 5 females and 5 males) included in the seminal CVC study [25] were used for statistical comparisons. This study had the prior approval by the ULB-Hôpital Erasme Ethics Committee. The subjects gave written informed consent before participating to the study.

### Experimental paradigm

The experimental paradigms used in this study were adapted from [25]. Subjects' cortical neuromagnetic signals were recorded while they were listening to live (*Live*) or recorded (*Recorded*) voices continuously reading a text in French during 5 min. Both texts were chosen for their emotional neutrality in order to reduce the effect of affective prosody, and for the absence of any dialog in order to minimize prosody modulations. As in the seminal study, the first condition (*Live*) consisted in the passive listening of a text (http://www.eodi.org/la_revolution_francaise.html) read by a native French-speaking male (XDT, same as Exp1m in [25]) sitting 2 m in front of the subject inside the magnetically shielded room (MSR). The reader's vocal activity was recorded, time-locked to MEG signals, with a three-axis accelerometer attached to the left side of the reader's throat (ADXL330 *i*MEMS Accelerometer, Analog Devices, Inc., Norwood, MA, USA). Subjects were asked to fixate the gaze at a point in the MSR to avoid any gaze contact with the reader. In a second condition (*Recorded*), subjects passively listened to a recording of a native French-speaking female reading a French text (http://www.litteratureaudio.com/livre-audio-gratuit-mp3/zola-emile-la-terre.html) during 5 min. This condition was designed to test the influence of human interactional factors (e.g., human presence) on the CVC phenomenon. A loudspeaker (Panphonics Oy, Espoo, Finland) was positioned 2 m in front of the subjects inside the MSR. Signals of the recorded voice were recorded time-locked to MEG signals. In a third 5-min session (*Rest*), subjects were asked, as in the two previous conditions, to relax, fixate the gaze at a point in the MSR and not to move. The three experimental conditions (*Live*, *Recorded*, and *Rest*) were randomized across subjects.

### Data acquisition, preprocessing, and analyses

The methods used for data acquisition, preprocessing, and analyses have been extensively detailed in [25] and will therefore be briefly described below.

#### Data Acquisition

MEG signals were recorded at the ULB-Hôpital Erasme with a whole-scalp-covering neuromagnetometer (Vectorview & Maxshield™; Elekta Oy, Helsinki, Finland). Head position inside the MEG helmet was continuously monitored using four head-tracking coils. The locations of the coils and at least 150 head-surface (on scalp, nose and face) points with respect to anatomical fiducials were determined with an electromagnetic tracker (Fastrak, Polhemus, Colchester, VT, USA). The recording passband was 0.1–330 Hz for MEG signals and 0–330 Hz for accelerometer and recorded voice signals; all signals were sampled at 1 kHz.

#### Data preprocessing

Continuous MEG data were preprocessed off-line using the signal-space-separation method to suppress external interferences and to correct for head movements [37]. In one subject, MEG signals in *Live* were preprocessed off-line using the spatio-temporal signal-space-separation (coefficient correlation: 0.95, window length: 4 s) to subtract system artefacts. Then, for frequency and coherence analyses, 2048 ms epochs were extracted with 1638 ms overlap, leading to a frequency resolution of 0.5 Hz. Epochs where MEG signals exceeded 3 pT (magnetometers) or 0.7 pT/cm (gradiometers) were rejected to avoid contamination of the data by eye movements, muscle activity, or artefacts of the MEG sensors. This yielded more than 590 artifact-free epochs for each subject and condition.

#### Coherence analysis in sensor space

Subsequently, voice signals were band-passed around voice fundamental frequency (f0; 100–200 Hz, *Live*; 130–300 Hz, *Recorded*) and rectified in order to compute for each epoch the coherence between the f0 time-course and MEG signals. In both conditions, the analyses were based on the voice fundamental frequency (f0) as this parameter is a highly relevant feature for speech processing (see [25] for more details). The coherence is an extension of Pearson correlation coefficient to the frequency domain, quantifying the degree of coupling between two signals, providing a number between 0 (no linear dependency) and 1 (perfect linear dependency) for each frequency [38]. The degree of coupling between MEG signals (frequency band: 0.1–330 Hz) and the f0 time-course were first determined by computing coherence spectra in the sensor space. Frequencies that displayed significant coupling in the sensor space in most subjects were identified and used as frequencies of interest for coherent source analyses.

#### Coherence analysis in source space

Coherence analyses were then performed in the source space using the Montreal Neurological Institute (MNI) template. Notwithstanding the risk of decrease in spatial accuracy, the MNI template was used rather than individual structural magnetic resonance imaging (MRI) in order to facilitate ASD subjects' recruitment as some of them refused to undergo MRI. For each subject, MEG and segmented MNI coordinate systems were coregistered using the three anatomical fiducial points for initial estimation and the head-surface points to manually refine the surface coregistration. The MEG forward model was then computed using the MNE suite (Martinos Center for Biomedical Imaging, Massachusetts, USA). Cortical sources coherent with the f0 time-course were identified using dynamic imaging of coherent sources (DICS) [39] and subsequently visualized on the MNI template. Separate coherence maps were computed for each possible combination of frequencies of interest, subjects, and conditions. Both planar gradiometers and magnetometers were simultaneously used for inverse modeling.

Finally, to produce coherence maps at the group level, we computed the generalized *f*-mean across subjects of normalized maps, according to 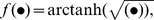 namely, the Fisher z-transform of the square root.

#### Statistical analyses

The statistical significance of sensor-space individual-level coherence was assessed using surrogate data-based statistics, based on coherence computed between surrogate f0 time-courses and original MEG data [40] (for more details, see also [41], [42]).

The statistical significance of source-space group-level coherence was assessed using a non-parametric permutation test based on coherence between vocal signals and rest condition MEG data [43] (for more details, see also [44]).

Non-parametric Wilcoxon and Mann-Whitney test were used to assess the difference between conditions and between ASD and typically developped subjects from the seminal CVC study [25]. Finally, correlations were computed to establish relations between coherence levels and age, and behavioural data reflecting autistic symptoms severity.

## Results

### Coherence at the sensor level

**Figure 1:**
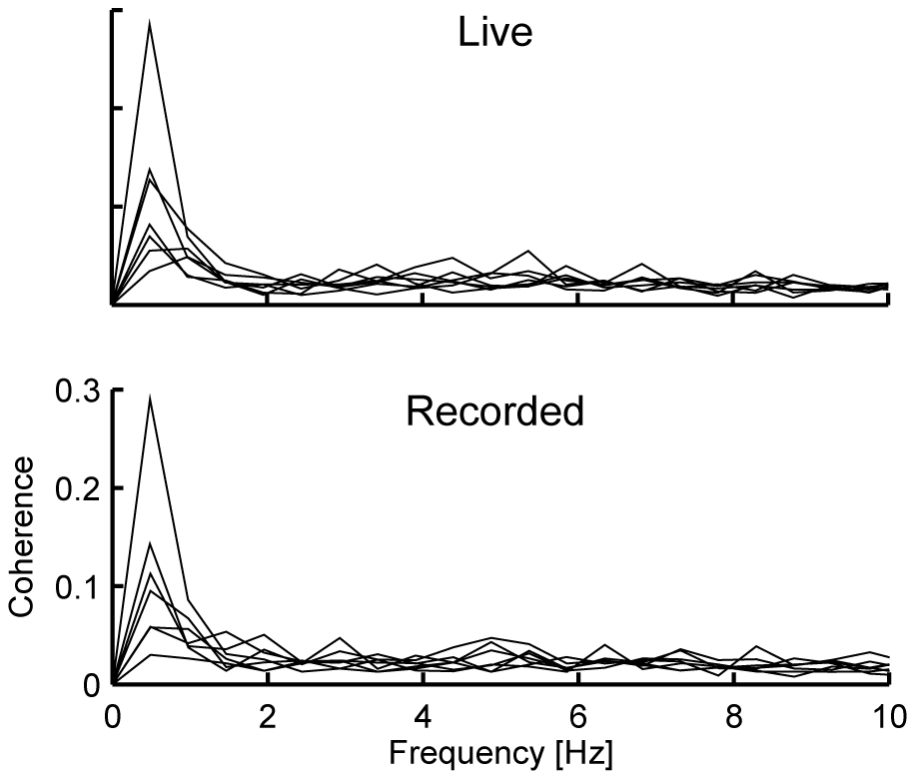
Individual coherence spectra in ASD subjects. Individual coherence spectra obtained in each condition for the right-hemisphere MEG sensor showing the maximum coherence level. Significant coherence at 0.5 Hz was observed in all ASD subjects in *Live* and in 6 out of 7 subjects in *Recorded*. Y–axis values are identical on both plots.

Figure 1 and Table 2 summarize the results obtained at the sensor level.

**Table 2:** Sensor-space coherence levels at 0.5 Hz.

Participants	Live	Recorded	Controls
1	0.1266	0.1130	0.0818
2	0.0692	0.0295*	0.0804
3	0.0804	0.0577	0.1302
4	0.0482	0.0953	0.0518
5	0.2846	0.2904	0.1647
6	0.0564	0.0582	0.1862
7	0.1377	0.1427	0.1255
8	—	—	0.0960
9	—	—	0.3072
10	—	—	0.1802
min	0.0482	0.0295	0.0518
max	0.2846	0.2904	0.3072
mean	0.1126	0.1121	0.1404

*p>0.05.

Live and Recorded: sensor-space coherence levels obtained in ASD patients.

Controls: sensor-space coherence levels obtained in Exp1m in the seminal CVC study (see Table 1, Bourguignon et al. 2013).

In line with the seminal CVC study carried out in a group of healthy adult subjects [25], coherence between voice and MEG signals consistently peaked at ∼0.5 Hz in all ASD subjects. Right-hemisphere-dominant coherence levels were statistically significant at ∼0.5 Hz in all subjects in *Live* (coherence values from 0.04 to 0.28; *p*<0.05; see Figure 1 and Table 2) and in six subjects in *Recorded* (coherence values from 0.06 to 0.29; *p*<0.05; see Figure 1 and Table 2). No difference in sensor-space coherence levels at 0.5 Hz was found between *Live* and *Recorded* (*p* = 0.87), nor between ASD subjects and the typically developed healthy adult subjects investigated in the seminal CVC study (*Live, p* = 0.28; *Recorded*, *p* = 0.31).

No other peak in coherence was found at the sensor level, and particularly at 4–6 Hz. The 0.5-Hz coherent frequency was therefore considered as the only frequency of interest to compute individual and group-level coherence maps.

### Coherence at the source level

**Figure 2:**
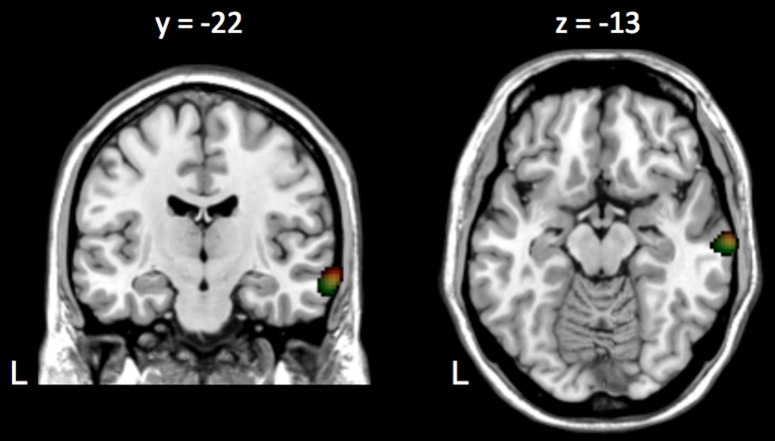
Group-level coherence maps in ASD subjects. Group level coherence maps computed at 0.5(*Live*, red; *Recorded*, green; intersection between *Live* and *Recorded*, yellow). In *Live*, the maximum coherence occured at the right superior temporal sulcus and in *Recorded*, at the upper bank of the middle temporal gyrus. Coherence maps were thresholded at the maximum of coherence to isolate the local coherence maxima (*Live*, coherence threshold: 0.64–0.71; *Recorded*, coherence threshold: 0.60–0.67).

Figure 2 illustrates the location of the coherence local maxima on the MNI template in both conditions.

Group-level analysis revealed maximum coherence at the right STS (MNI coordinates: [64 –15 –11] mm; coherence value: 0.07, *p*<0.05) in *Live* and at the middle temporal gyrus (MTG) (MNI coordinates: [64 –18 –18] mm; coherence value: 0.07, *p*<0.05) in *Recorded*. Considering the limited spatial resolution afforded by the coregistration procedure used in this study, these coherence local maxima can be considered very close to the one reported in the seminal CVC study (Exp1m, pSTS, [66 -26 -1] mm) [25].

No local coherence maximum was observed at supratemporal auditory cortex bilaterally. At a lower threshold, a local coherence maximum was also observed at the left parietal operculum in both listening conditions, in accordance with the seminal CVC study [25].

### Correlations between sensor-space coherence level and behavioural data

Correlation analyses did not disclose any association between sensor-space coherence levels and age or behavioural scores (age, IQ, ADOS and TLOCC: *p*>0.05).

## Discussion

Despite scientific evidences of right pSTS dysfunction in ASD, this study demonstrates significant coupling in ASD subjects between neural activity at this cortical structure and the slow fluctuations of a heard voice during continuous connected speech listening, a phenomenon previously demonstrated in healthy subjects. Indeed, the coupling between the reader's voice f0 time-course and listener's cortical MEG signals was similar at 0.5 Hz in terms of coherence levels and location of coherence local maxima between ASD subjects and the typically developed healthy adult subjects investigated in the seminal CVC study [25]. This study also suggests that CVC levels at 0.5 Hz do not vary according to age or autistic symptom severity in ASD subjects. No coupling was found at 4–6 Hz between the reader's voice f0 time-course and listener's cortical MEG signals in ASD subjects, in accordance with the scarcity of such coupling in typically developed subjects using our experimental setting [25].

### Preserved coupling between the reader's voice and listener's MEG signals in ASD

The preserved coupling between the reader's voice and listener's MEG signals observed in the ASD subjects investigated in this study may be explained by several factors. In line with the neural complexity hypothesis [45], [46] and the enhanced perceptual theory (for a review, see [47]), the cortico-vocal coupling might be preserved in ASD subjects because of the low-level neural processing engaged in this coupling phenomenon. Indeed, the CVC experimental paradigm consists in unimodal (auditory) stimulation. Moreover, this coupling phenomenon occurring at the right pSTS is considered to involve prelexical processing levels in speech perception as it was also observed when typically developed subjects listened to a totally incomprehensible language (native French-speaking subjects listening to Finnish) [25]. Interestingly, accumulating data suggest that ASD subjects perform normally, or even better than typically developed subjects, in tasks that require low-level processing or involve simple material [48], whereas they underperform in tasks that engage high-level neural processing such as multimodal sensory processing [49], [50] or complex cognitive or social tasks [13], [51]. Electrophysiological and neuroimaging data support this particular perceptive profile (for reviews, see [28], [46], [47]), which seems to be explained by an association of local over-connectivity and long-range under-/dys-connectivity [52], [53]. Still, some authors consider that speech perception is complex *per se* and numerous studies have suggested more severe impairments or cerebral dysfunctions for speech than for non-speech stimuli in ASD subjects [28], [46]. However, albeit speech signals are indeed highly complex auditory stimuli, it might be hypothesized that the prelexical speech perception processes subtending the CVC phenomenon involve low-level neural processes that would not be impaired in ASD subjects.

It could be hypothesized that the preserved coupling observed in our ASD subjects might have been favored by the slow rate at which the suprasegmental pauses or silences occur in speech (typically, for sentence rhythmic prosody, below 1 Hz). Indeed, it has been proposed that the temporality of the sensory stimulations, apart from their multimodal character, plays a key role in the pathophysiological basis of some behavioural impairments characterizing ASD [53]. Indeed, although altered temporal integration in the auditory modality as well as impaired spatio-temporal integration in the visual modality have been repeatedly observed in this population, slowing down the speed of sensory stimulations has been shown to improve imitative, verbal and cognitive performances [53]. It might therefore be of interest to determine if the coupling observed for shorter timescales (4–7 Hz, a TW corresponding to syllables and some prosodic phenomena) between neural and vocal activities during speech are preserved or not in ASD subjects [54].

Finally, a functional MRI study investigating the neural basis of prosodic speech deficits (linguistic and emotional prosody) in subjects with high functioning autism failed to find any clear deficit in pSTS recruitment compared with typically developed healthy subjects [55]. But and more interestingly, apart from an increased recruitment of the left supramarginal gyrus, ASD subjects were characterized by an absence of deactivation in key nodes of the default mode network (DMN) such as the precuneus, the anterior cingulate cortex and the left middle frontal gyrus; DMN deactivation correlating with prosodic skills in that study [55]. The DMN is a set of brain regions characterized by a high level of metabolic activity at rest and a low level of activity during goal-directed or externally focused cognitive tasks (for a review, see [56]). Several lines of evidence suggest that DMN suppression during goal-directed tasks is functionally relevant, particularly in terms of cognitive performance (i.e., the more the DMN activity is suppressed, the better is the cognitive performance) (for a review, see [57]). DMN suppression possibly reflects adaptive disengagement of “goal-irrelevant” brain functions (e.g., mind-wandering), which would be required to get properly engaged in goal-directed tasks [57]. So, the inability of deactivating DMN nodes while processing prosodic connected speech observed in ASD subjects could account for the existence of a less efficient processing of the relevant information, i.e. the prosodic dimension of speech, in ASD subjects [55]. Whether the deficits in DMN deactivation are related to altered functional integration between task-dependent networks and the DMN, or to a DMN dysfunction per se remains an open issue. Still, these fMRI data are congruent with the results of the present study suggesting preserved neural processing of suprasegmental rhythmic prosody at the right pSTS. Taken together, these data suggest that alterations in functional integration beyond the pSTS might therefore account for the rhythmic prosodic impairments described in the ASD population. This latter hypothesis is supported by MEG data obtained in subjects with Asperger syndrome that investigated another sensory modality, i.e. social visual cues processing [58]. In that study, neural activations were normal in strength and timing at the early stage of the cortical activation sequence (i.e., visual, STS and inferior parietal lobule activations) but were abnormal for the later stage (i.e., inferior frontal and primary motor cortex activations). This finding was interpreted as the absence of any significant deficit of high-level visual stimuli processing at the STS level in Asperger syndrome [58].

Importantly, the preservation of early cortical processing of prosodic elements in verbal language might potentially be exploited in therapeutic interventions in ASD. An anecdotic report already suggests in 1979 that the stress put on the melodic content of language has been helpful in the language therapy of an autistic boy [59]. The benefit of music therapy for verbal communication reported in ASD might also stem from the low frequency variations of auditory (music) stimuli carrying pertinent information potentially exploitable for social communication improvements [60], [61]. Also, it is important to consider that disruption of interactional synchrony is an important feature of ASD that has recently attracted some attention [62]. Since interpersonal synchrony is the dynamic and reciprocal adaptation of the temporal structure of behaviors between interactive partners [63], preservation of coherent activity at the pSTS suggests that a neural substrate is present in ASD for interactional synchrony based on auditory inputs during dyadic oral communication. This represents a neurophysiological support for early therapeutic interventions based on prosody, including those building on the typical prosodic characteristics of the so-called “parentese”, a special type of speech directed towards infants typically used by adults (higher pitch, slower tempo, and exaggerated intonation contours [62].

### Limitations of the study

First, ASD subjects were compared with the typically developed healthy subjects investigated in the seminal CVC study [25] and were therefore not matched for age and sex. Still, as all ASD subjects (except for one subject in *Recorded*) obtained significant sensor-space coherence levels with values similar to those observed in the seminal CVC study, we consider that the conclusions of this study are not affected by this limitation.

Second, the use of the MNI template for the source space coherence analyses in ASD subjects limits the accuracy of the coherent source localization (as compared with the use of individual MRI) [64]. Still, in the absence of an individual MRI, using a template head model results in a fairly precise head model for source space modelling [65]. This is further supported by the fact that, even with this lower spatial resolution approach, the localization of the right STS maximum coherence was very close to the one described in the seminal CVC study. This finding highly suggests that similar brain areas are involved in the CVC phenomenon in ASD and typically developed subjects.

Finally, we did not test behaviourally the rhythmic prosody perception skills of our ASD subjects, which limits the interpretation of the functional relevance of the preserved CVC phenomenon in this population. Indeed, even if our result highly suggest a normal neural processing of the suprasegmental speech rhythmic prosody at the prelexical level, we cannot determine if this preserved coupling gives rise to normal prosody perception at the behavioural level. The ASD subjects included in this study had weak to clear expressive prosodic disorder. This highlights the importance to investigate in future studies the correlation between CVC levels and prosody perception skills.

### Conclusions

This study discloses a preserved coupling between the reader's voice and the listener's cortical activity in ASD subjects at the right pSTS. These findings support the existence of preserved neural processing of sentence-level rhythmic prosody in ASD. The preservation of early cortical processing of prosodic elements in verbal language might be exploited in therapeutic interventions in ASD.
